# Design and Fabrication Technology of Low Profile Tactile Sensor with Digital Interface for Whole Body Robot Skin

**DOI:** 10.3390/s18072374

**Published:** 2018-07-21

**Authors:** Mitsutoshi Makihata, Masanori Muroyama, Shuji Tanaka, Takahiro Nakayama, Yutaka Nonomura, Masayoshi Esashi

**Affiliations:** 1 Department of Robotics, Tohoku University, Miyagi 980-8579, Japan; tanaka@mems.mech.tohoku.ac.jp; 2 Microsystem Integration Center, Tohoku University, Miyagi 980-8579, Japan; muroyama@mems.mech.tohoku.ac.jp; 3 T-Frontier Div., Toyota Motor Corporation, Toyota, Aichi 470-0309, Japan; takahiro_nakayama_aa@mail.toyota.co.jp; 4 Toyota Central R&D Labs., Inc., Aichi 480-1192, Japan; nonomura@meijo-u.ac.jp

**Keywords:** tactile sensor, MEMS-CMOS integration, wafer level packaging, sensor network, through silicon via, benzocyclobutene

## Abstract

Covering a whole surface of a robot with tiny sensors which can measure local pressure and transmit the data through a network is an ideal solution to give an artificial skin to robots to improve a capability of action and safety. The crucial technological barrier is to package force sensor and communication function in a small volume. In this paper, we propose the novel device structure based on a wafer bonding technology to integrate and package capacitive force sensor using silicon diaphragm and an integrated circuit separately manufactured. Unique fabrication processes are developed, such as the feed-through forming using a dicing process, a planarization of the Benzocyclobutene (BCB) polymer filled in the feed-through and a wafer bonding to stack silicon diaphragm onto ASIC (application specific integrated circuit) wafer. The ASIC used in this paper has a capacitance measurement circuit and a digital communication interface mimicking a tactile receptor of a human. We successfully integrated the force sensor and the ASIC into a 2.5×2.5×0.3 mm die and confirmed autonomously transmitted packets which contain digital sensing data with the linear force sensitivity of 57,640 Hz/N and 10 mN of data fluctuation. A small stray capacitance of 1.33 pF is achieved by use of 10 μm thick BCB isolation layer and this minimum package structure.

## 1. Introduction

Whole body tactile sensation is a desired function in the current movement of robots into our society to assist humans. In contrast to industrial robots, these robots are required to work safely in an uncontrolled environment and to have highly versatile capabilities of action [1]. There are a tremendous amount of studies and products that have been developed in the past thirty years. However, the majority of these tactile sensors are not considered to cover a whole body of a robot but designed to achieve the best performance as a single chip such as multi-axis sensing, high-density array, and flexibility [2]. The recent requirements for the whole body tactile sensor are a capability for large area sensing, satisfaction of both large number of sensor and rapid response time, and low cost. Here, MEMS (Micro Electro Mechanical Systems) technology would fulfill these requirements as miniaturization of both mechanical force sensor and electronics can be realized by the wafer scale microfabrication process at a low cost.

**Figure 1:**
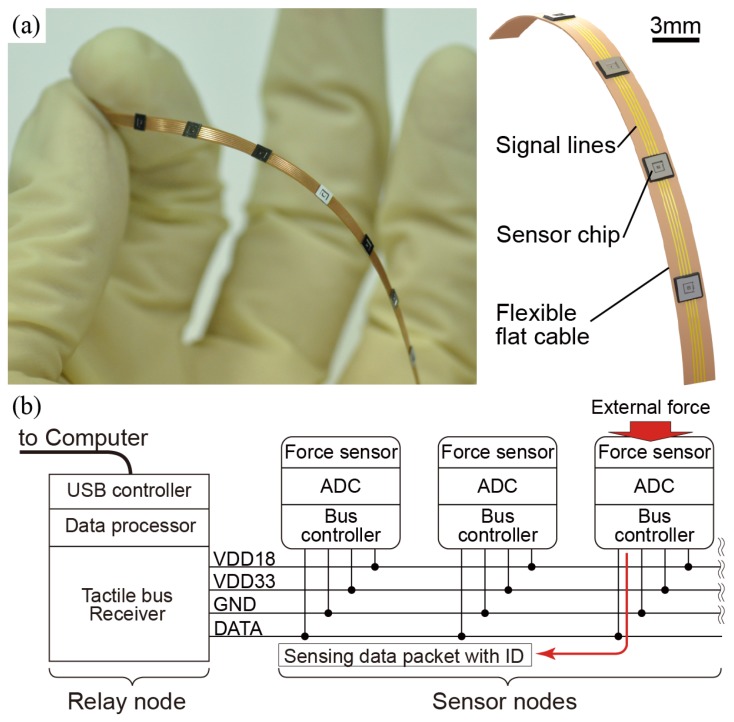
A tactile sensor network with small and smart sensor dies: (**a**) one-dimensional sensor array on a flexible cable; (**b**) tactile sensors with a force sensor and bus network interface on a chip.

We have proposed MEMS tactile sensors which have a mechanical force sensor and an integrated circuit to realize the whole body tactile sensation by forming a serial network. Figure 1 shows the overview of the tactile sensor system. Tactile sensors which contain a force sensor, Analog–Digital converters (ADCs), and digital communication interfaces are mounted on a flexible cable with a serial bus line (Figure 1b). Each tactile sensor is designed to be able to judge whether a sensor is pressed or not according to the specific threshold value. This autonomous protocol is mimicking the tactile receptor in a human body fire nerve pulses when stimulus is meaningful. This protocol will mitigate the decrease of time resolution as the number of sensor increases and would satisfy all the requirements mentioned above. On the other hand, as shown in Figure 1a, sensors must be packaged as a surface-mountable and a low-profile and low-cost packaging. It is challenging and satisfying both the sensor–electronics fusion and an advanced packaging at the same time has barely been studied. Our previous studies revealed that the critical factors for the successful prototyping are the design of a chip structure and a manufacturing process [3,4,5,6].

In this paper, we present design, fabrication technologies and evaluation of a newly developed tactile sensor. Section 2 describes designs of a package, force sensor and a digital interface on the premise that a tactile sensor can be manufactured in a feasible manufacturing process. In Section 3, details of a fabrication process used for prototyping are presented, which utilized our unique low-cost through silicon interconnection and hetero wafer bonding technology. Finally, the fully functional dies (1.7 mm${}^{3}$ in volume) are prototyped, and a response to an external force and autonomous packet transmission are confirmed (Section 4).

## 2. Design of Tactile Sensor

### 2.1. Package and Device Structure

The major premise of the design of the tactile sensor is the application of a wafer bonding technology to integrate a mechanical sensor and electronics. Ordinary monolithic integration of a MEMS and CMOS (Complementary Metal Oxide Semiconductor) device involves the migration of a manufacturing process which causes restriction on performance, cost and development speed. On the other hand, the MEMS-CMOS integration using wafer bonding of separately manufactured wafers allows for using ASIC wafers produced by the latest CMOS process to be integrated with a MEMS wafer optimized and manufactured independently [7,8,9].

**Figure 2:**
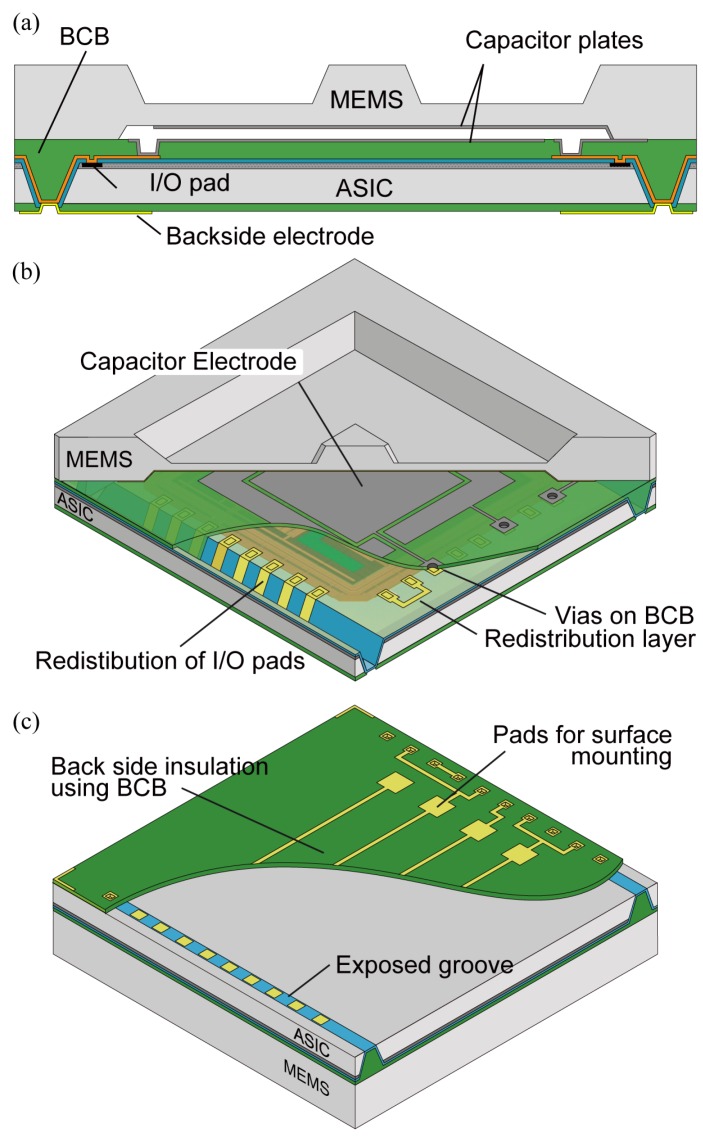
Device structure: (**a**) cross section of the chip; (**b**) top side; (**c**) back side.

Figure 2 shows the structure of the device that satisfies the requirement of the surface mount device package and wafer level packaging. A silicon diaphragm structure is stacked on the ASIC wafer by using an intermediate adhesive layer of BCB polymer, which is widely used in wafer bonding and the packaging industry. A pair of electrodes between the MEMS diaphragm and a BCB layer is to sense an external force through a capacitance change. Figure 2b shows more detailed features of the device. Two tapered grooves are formed near the edge of a die which works as through silicon interconnection. The redistribution layer of I/O pads penetrates along grooves whose bottom are exposed at the backside of a chip. We named this structure “Through Silicon Groove (TSG)” after the through silicon via (TSV). In this structure, a MEMS diaphragm encapsulates the surface of ASIC. Therefore, integration and packaging are done simultaneously. In this structure, the BCB polymer plays several important roles, which are filling grooves, electrical isolation of capacitive electrode from an active layer of ASIC, and an adhesive for wafer bonding. The reason for the use of the BCB polymer is because it has ideal properties for wafer bonding and packaging such as low outgassing and small volume shrinkage (<5%) during a polymerization. The cured BCB has high glass transition temperature (>350 ${}^{\circ }$C) and moderate chemical resistivity for solvents which expand a choice of processes after bonding [10,11].

### 2.2. Quasi Linear Force Transducer

**Figure 3:**
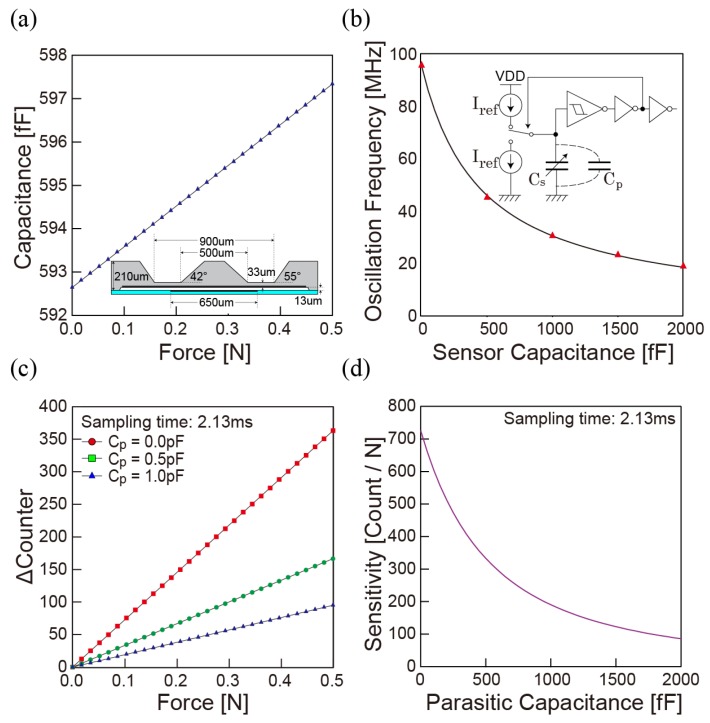
Mechanical and circuit simulation: (**a**) mechanical simulation of the force sensing element; (**b**) capacitor–frequency simulation extracted from the layout of Schmitt trigger oscillator in the ASIC; (**c**) calculated change of digital counter under an external force on a diaphragm; (**d**) influence of parasitic capacitance on sensitivity.

Among many studies of a MEMS-based tactile sensor, the capacitive force sensor made with a silicon diaphragm is chosen for further miniaturization as it needs a minimum number of electrical interconnections between the MEMS and ASIC wafer. We design the silicon diaphragm that can stand a vertical force up to 30 N for heavy-duty use by introducing a thick diaphragm. The small deformation of diaphragm can be detected by measuring a capacitance between electrodes formed at the air gap. The relation between an external force and capacitance is calculated using COMSOL Multiphysics as shown in Figure 3a. The linear response up to 0.5 N is achieved due to the thick diaphram design and the simulation also confirms that mechanical stress is below 4 GPa when the diaphragm contacts to bottom.

The principle of the capacitor sensing is based on capacitance to frequency (CF) conversion by combining a constant current source and Schmitt trigger circuit as shown in the inset of Figure 3b. The relation of oscillation frequency ($f_{shmt}$) sensor capacitor ($C_{s}$) can be written as Equation (1), where $V_{SPH}$ and $V_{SPL}$ are upper and lower voltage thresholds of the Schmitt trigger, respectively, and $I_{REF}$ is constant current to charge and discharge the capacitance. $C_{p}$ is parasitic capacitance typically formed in parallel with $C_{s}$. The frequency of the oscillator is digitalized by counting edges of the oscillation signal in given sampling time ($T_{sample}$) and numerical offset as written in Equation (2). Combining mechanical and electrical simulation above results in the linear correlation between digitalized frequency value and external force as shown in Figure 3c. This is because of a very small capacitance change of MEMS force sensors. On the other hand, the sensitivity varies drastically as a parasitic capacitance $C_{p}$ increase due to the nonlinearity of a CF converter with capacitance. Figure 3d indicates the relation between sensitivity and parasitic capacitance and shows the importance of the reduction of parasitic capacitance:

(1) $$f_{shmt}=\frac{I_{REF}}{2\left(C_{s}+C_{p}\right)\left(V_{SPH}-V_{SPL}\right)}$$

(2) $$counter=offset-f_{shmt}\times T_{sample}.$$

### 2.3. Overview of the ASIC

In addition to the CF conversion circuit mentioned above, the ASIC developed for this study has a high versatility to increase the capability of sensing. The sensing parameters such as sampling rate, offset and threshold values are stored in a volatile memory embedded in ASIC, which can be programmed by serial communication (Figure 1b). As this communication is designed to happen only when the power line is turned on, this procedure is called initialization. The digital circuit running with a 45 MHz on-chip clock based on the RC oscillator enables those logical functions mentioned above.

Design of a communication protocol is the most critical factor in determining the performance of a tactile sensor as a system. We have proposed the unique serial communication protocol mimicking the tactile receptor in our skin that transmits a nerve signal only when stimulus exceeds a certain threshold [12,13]. In contrast to a conventional serial bus network, this system uses the simplex communication to receive meaningful data among many sensors by letting sensors transmit when data exceed the threshold. The protocol we developed can be categorized to 1-persistent CSMA (Carrier Sense Media Access) protocol in which each sensor with valid data keeps checking the traffic of bus and send packets immediately once the absence of packets is detected. As the information in packets is independent of each other, collision detection is not implemented on a sensor side to maximize the throughput of this system. The packet used for this communication contains a header and footer, 8-bit ID number which is defined by fusing I/O pads at a packaging process, 32-bit sensing data from CF converter and a 16-bit CRC (Cyclic Redundancy Check) code to secure the data reliability. These 68-bit data are encoded into 85-bit with 4B/5B and NRZI (Non-Return to Zero Inverted) for asynchronous digital communication using clock data recovery. Our practical experiences have expected that maximum bandwidth of this protocol would be 35 Mbps if packet collisions between more than three chips are ignored [13], which allows 255 sensors to transmit 1600 packets every second. In other words, the maximum delay time is about 620 μs in the worst case in which all sensors are pressed at a same time.The thresholding in packet generation will drastically improve the response time.

## 3. Fabrication Process

### 3.1. Through Silicon Groove (TSG) Technology

**Figure 4:**
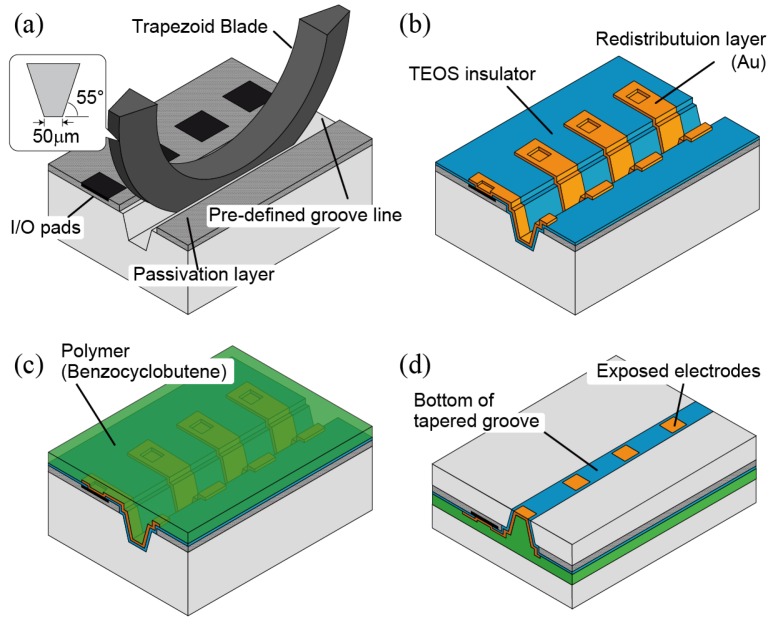
Procedure of the through silicon groove (TSG) technology: (**a**) groove forming; (**b**) insulation and rewiring of I/O pad into groove; (**c**) groove filling with polymer and planarization; (**d**) back grinding.

Tactile sensing has the principle that physical contact is involved, which makes the TSV technology essential as packages must have electrodes on the back and sensing surface on the front of a chip. Our original technology, TSG, uses the mechanically formed grooves by the dicing technique instead of an etching process. In contrast to TSV [14], TSG can easily control taper angle by the shape of a dicing blade and achieve a high-throughput and low-cost manufacturing of through silicon interconnections. Figure 4 indicates the procedure of TSG forming. A groove forms the area next to a scribing area, while another area is protected from contamination by photoresist coating. This photoresist also works as a masking layer for the etching of a passivation layer to avoid chipping at the edge of the groove. In this prototyping, the diamond blade manufactured by Disco Corporation (Tokyo, Japan), “B1A series (55${}^{\circ }$ tapered, 50 μm flat tip, #400 grit size)” is used (Figure 4a). Wafer fixing using wax instead of a dicing tape is preferable to avoid unevenness of a depth of grooves. The depth of the groove is precisely controlled to be 70 μm. Followed by atomized jet spray cleaning with ethanol [15], a groove is insulated by the low-temperature oxide layer (Tetraethyl orthosilicate: TEOS) deposited by PECVD (Plasma Enhanced Chemical Vapor Deposition). After the oxide layer covering I/O pads is removed, a few micrometers of Au redistribution layer is electroplated to extend I/O pads to the bottom of a groove (Figure 4b). The lithography process with a deep groove requires a spray coating of photoresist. BCB polymer is filled into grooves and flattened for the following wafer bonding process (Figure 4c). After bonding with a MEMS wafer, ASIC side is thinned by a back-grinding to expose electrodes at the bottom of a groove (Figure 4d).

### 3.2. Polymer Processing and Wafer Bonding

**Figure 5:**
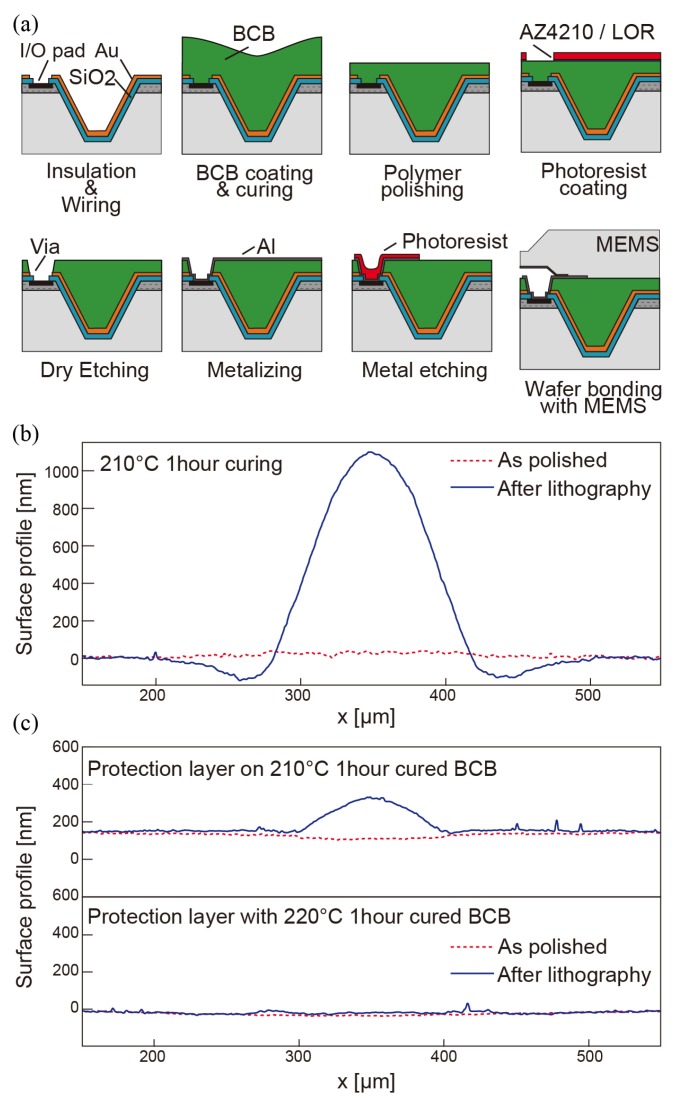
Filling of grooves with BCB and anti-swelling layer: (**a**) procedure of TSG forming; (**b**) swelling at groove due to swelling of BCB; (**c**) suppression of swelling by anti-swelling layer.

The technological challenge of this device structure is the preparation of an intermediate polymer layer between MEMS and ASIC wafer. This polymer layer works as a filling material of the groove, electrical isolator for a capacitive electrode from ASIC wafer, and adhesives for wafer bonding. Furthermore, this polymer layer has to be mechanically rigid enough while bonding to maintain the air gap with a silicon diaphragm, capacitor electrodes and via holes. There are many studies about wafer bonding technology with a patterned BCB layer as an application for wafer level packaging. These studies show that BCB has unique properties and that partially cured BCB has enough mechanical and chemical strength for its processing while it still has an adhesion for wafer bonding [16,17,18]. However, this prototyping requires more complex and various processes between a coating of polymer and a wafer bonding such as photolithography, metal deposition, and polishing. In this prototyping, we succeeded in the process integration satisfying these requirements by introducing a mechanical polishing of BCB polymer, the anti-swelling technique of BCB, and a polymerization ratio control. The details of the fabrication process focusing on polymer processing is illustrated in Figure 5 and described below.

First, grooves are filled with BCB by an ordinal spin coating of a BCB solution (Cyclotene 3000-63 from Dow Chemical Company (Midland, MI, USA)). The tapered shape helps to fill the groove without bubbles. After curing coated polymer up to a certain polymerization degree in an inert gas environment, the BCB film is polished using Logitech’s LP50 precision polishing machine (Glasgow, UK) with aluminum slurry and surfactant additive to improve wettability. The details of this process are summarized in Table 1. The optimized polishing process achieves 2 nm of Ra and less than 30 nm waviness near grooves. Another important property of the polished surface is parallelism for wafer bonding. Fixing a wafer onto a highly parallel plate using low-temperature wax helps to obtain a 0.06${}^{\circ }$ of parallelism of the BCB film. Keeping a BCB surface flat while further processing is an essential factor to achieve void-less wafer bonding. As shown in Figure 5a, two photolithography processes, one metallization process, and one dry etching are required after polishing. Our experiments revealed that a severe swelling happens after lithography process with AZ® 4500 series photoresist (Microchemicals, Ulm, Germany), which causes large waviness at a groove area and prevents void-free bonding (Figure 5b). We believe the solvent of the photoresist, PGMEA (Propylene Glycol Monomethyl Ether Acetate) causes swelling of partially cured BCB. There, Coating of lift-off resist (LOR) from MicroChem Corp. (Westborough, MA, USA) right after the BCB layer is polished is introduced as a barrier layer for permeation of PGMEA into the BCB layer. As a result, waviness at grooves after photolithography is suppressed five times smaller (Figure 5c). Furthermore, increasing polymerization ratio by raising the temperature of half-curing improves flatness after photolithography. The introduction of the solvent barrier layer lowered the minimum requirement of the curing ratio of the BCB layer in consideration of the swelling issue. This finding enlarged the process windows.

A polymerization degree of the BCB on the polishing process is adjusted to be more than 50% to have chemical resistance to some extent. A BCB cured at 210 ${}^{\circ }$C for one hour has chemical resistance to Isopropanol, Ethanol, but acetone causes a deep crack after soaking for several minutes. The suitable polymerization degree will be between 50% and 75% to achieve void-free bonding and keep the shape of the BCB polymer. Our previous research confirmed that polymerization degree would not be changed by RIE (Reactive Ion Etching) and the photolithography process [18].

### 3.3. Detail of the Fabrication Process

**Figure 6:**
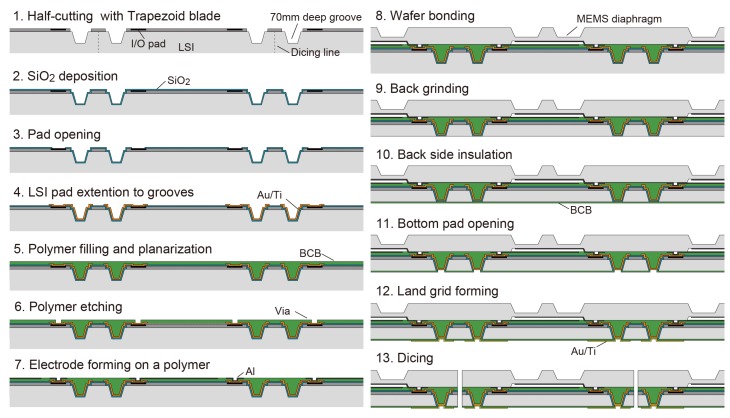
The full process chart of wafer level integration and packaging for tactile sensors.

The prototyping of the tactile sensor is done with the ASIC wafer manufactured by TSMC (Taiwan Semiconductor Manufacturing Company, Hsinchu, Taiwan) with 0.18 μm technology. Figure 6 shows the full details of the fabrication process. First, the tapered grooves for the TSG are formed by the tapered blade with a flat tip at the dicing street. The side wall is insulated by a 1 μm ${\mathrm{SiO}}_{2}$ layer deposited by TEOS-PECVD. The ${\mathrm{SiO}}_{2}$ deposited on I/O pads are removed by dry etching with spray coated photoresist. Then, a 5 μm thick redistribution layer of Au is electroplated towards the bottom of groove with Ti and Au seed layer, and grooves are filled with a partially-cured BCB polymer. After filling the grooves, BCB polymer is made flat enough for wafer bonding by mechanical polishing. The thickness of the BCB layer is controlled to be 10 μm by using an optical interferometer. Vias on the BCB layer are opened by $O_{2}$ + ${\mathrm{SF}}_{6}$ RIE with a photoresist mask, and then capacitor electrodes are formed by sputtering and etching of Al.

Next, the individually manufactured MEMS wafer is bonded onto the ASIC wafer using BCB as an adhesive. Silicon diaphragms with a protuberance are fabricated by anisotropic wet etching with the mixture of TMAH (Tetra Methyl Ammonium Hydroxide) (25 wt %, 80 ${}^{\circ }$C) and Triton-X100 (0.05 wt % to TMAH solution) for defect-free single crystal structure. Anti-surfactant in TMAH enables the forming of an island pattern [19]. Etching angle is measured to be 42${}^{\circ }$ due to side-etching and appearance of a different crystal plane. Wafer bonding is carried with 3.75 MPa under lumping of temperature from room temperature to 270 ${}^{\circ }$C within 10 min and holding both pressure and temperature for an hour. After wafer bonding, the bonded wafer is lapped and thinned down to 70 μm from the ASIC wafer side. ${\mathrm{XeF}}_{2}$ vapor etching of Si is used to expose grooves gently if necessary. The backside of the ASIC is then insulated by a 1 μm thick BCB layer. Vias are opened on the polymer insulator by RIE and the insulator at the bottom of a groove is etched simultaneously. The backside electrodes are prepared by electroplating of Au with Au/Ti seed layer. Finally, the wafer is fully diced into chips.

## 4. Evaluation of a Tactile Sensor

**Figure 7:**
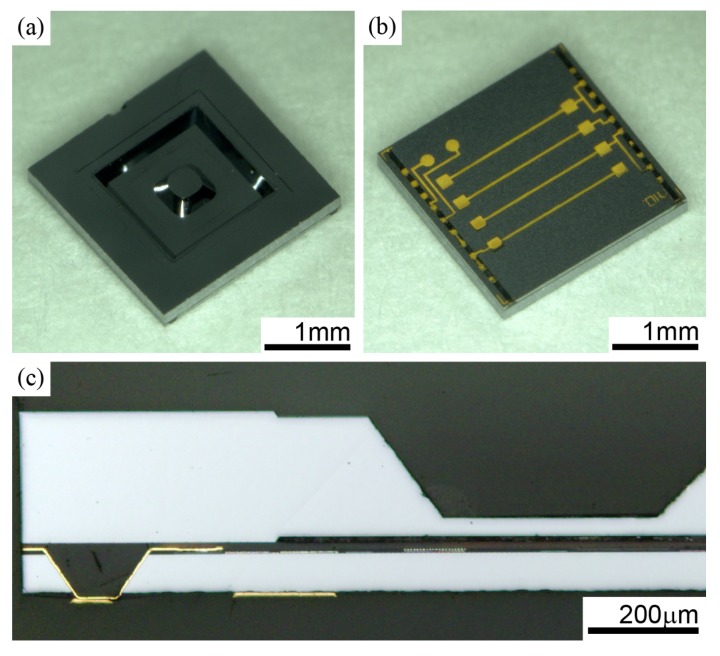
Prototyped tactile sensor: (**a**) front; (**b**) back; and (**c**) cross sectional view.

The pictures of the completed device are shown in Figure 7. The dimension of each die is 2.5×2.5×0.3 mm, which is 1.7 mm${}^{3}$ in volume. The package has a footprint the same as die size of the ASIC and an ultra-low profile is achieved by the back grinding. This package is ready for the mounting of flexible flat cables using surface mounting technology with an anisotropic conductive paste. As shown in Figure 7c, the microscopic cross-sectional image of die indicates that I/O pads are correctly penetrating to the backside among a tapered groove. In addition, the air gap between silicon diaphragm and ASIC is successfully formed, not collapsed by deformation of BCB layer due to the high pressure of wafer bonding. This is the result of an appropriate control of polymerization degree of BCB polymer before the wafer bonding process. The prototyped eight chips out of 12 chips are successfully operated. In addition, a yield of the TSG technology investigated by an open-short test with an LSI tester (Advantest T6573, Tokyo, Japan) is 71% (*n* = 84). Forming grooves before wafer bonding improves a yield significantly compared to the packaging method which forms through holes after bonding we have reported previously [6].

**Figure 8:**
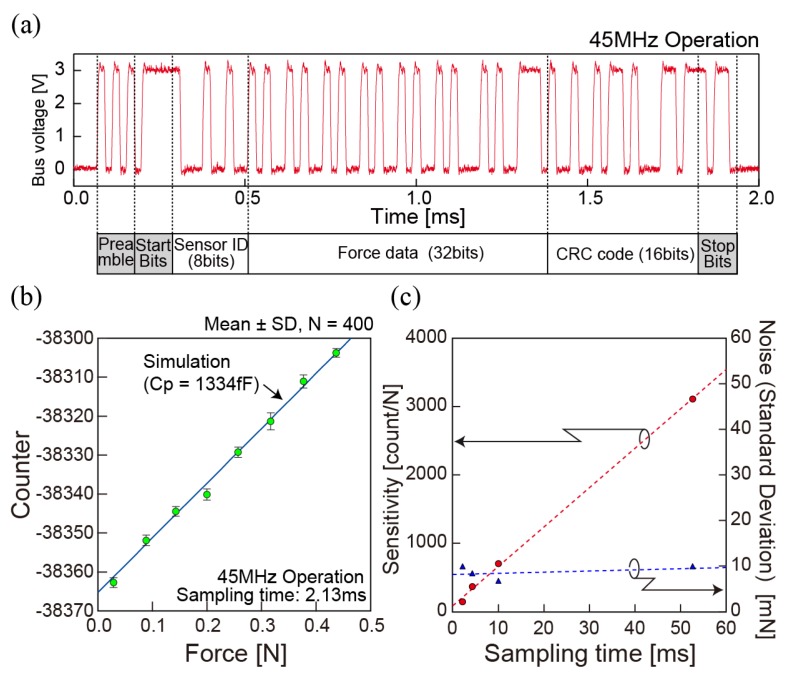
Experimental data with prototyped sensors: (**a**) a 45 MHz digital packet; (**b**) linear correlation between encoded counter value and external force; (**c**) sensitivity and data fluctuation with various sampling times.

The evaluation of completed chips is done with an FPGA (Field Programmable Gate Array) based data acquisition system specially designed to initialize sensors, receive digital packets on a sensor bus, and transfer the data to a computer. The 100 MHz local oscillator on the FPGA board enables the receiving of 45 Mbps asynchronous packets and precise measurement of packet arrival time. The performance of each sensor is evaluated by connecting a single chip to the receiver with the initial setting that allows the sensor to transmit packets as soon as capacitance is measured regardless of value. Figure 8a shows a packet spontaneously transmitted from a prototyped chip. The 32 bits of force data that represent the counter value from the CF converter is decoded and transferred to a computer through USB 2.0 (Universal Serial Bus, 480 Mbps bandwidth) with a timestamp measured on the FPGA board.

The linear response of the sensing data with external force is confirmed as shown in Figure 8b. By initializing the offset parameter and sampling time to zero and 2.13 ms, the parasitic capacitance introduced by the integration can be calculated from the counter data by comparing with the simulation results. Parasitic capacitance 1.33 pF calculated from the fitting is reasonable value compared to the estimated value considering 670 μm × 670 μm square electrode and the surface of ASIC separated by 10 μm of BCB film ($\epsilon_{r}$ = 2.7). The simulation results with this stray capacitance are well matched with the measured result (140.6 Count/N, $\chi^{2}=-3.67\times 10^{-4}$). The noise level is delivered from the data fluctuation, whose standard deviation of the signal is 1.4 Count, which is equal to 10 mN (*n* = 400 packets).

**Figure 9:**
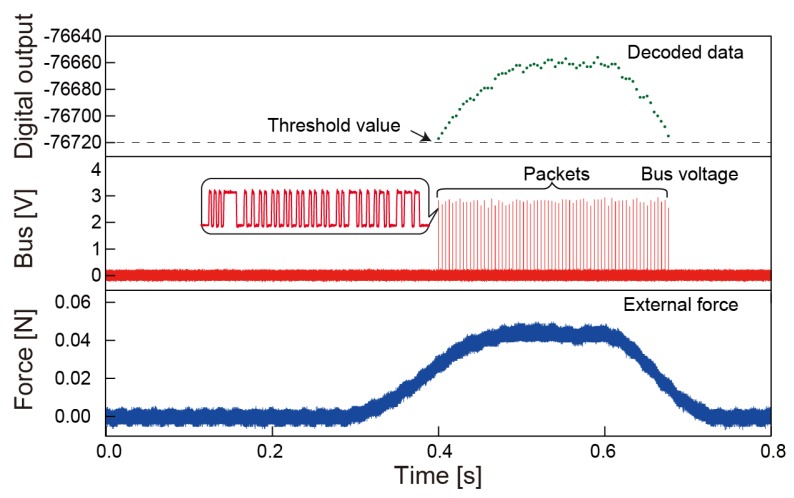
Response to an external force with threshold operation.

Signal-to-noise ratio is calculated to be 90 dB as designed maximum force is 30 N. Figure 8c shows sensitivity and noise level as a function of a sampling rate, which is tuned by the initialization when a chip powered up. By fitting sensitivity as a function of $T_{sample}$, generalized sensitivity of 57,640 Hz/N is obtained. Based on the fact that the noise level converted to the unit of a force does not decrease as sampling time increases, we believe that noise from capacitance is negligibly small and jitter of the on-chip clock is a dominant source of the data fluctuation as it defines accuracy of the frequency counting. Figure 9 shows the demonstration of event-driven packet transmission with threshold operation and impulsive stimulation on the chip. By using parameter setting on power, a single sensor is initialized so that it transmits packets only when sensing results exceed a specific value. A sampling time, an offset, and a threshold value are set as 4.25 ms, 0, and, −76,720, respectively. It is obvious that this event-driven protocol saves bandwidth by sending only meaningful data, and achieves 30% data compression in the time frame shown in the Figure.

## 5. Conclusions

In this paper, the concept of the tactile sensor chip with a communication function is proven by prototyping the new device chip composed of a silicon diaphragm and ASIC that are integrated and packaged by the batch fabrication. The through silicon interconnection by using mechanically formed grooves on ASIC is invented so that the tactile sensor can be installed on a bus line with surface mounting technology. In addition, the breakthrough in polymer processing using polishing and introduction of the anti-swelling layer of BCB polymer led this prototype to success. The chip packaged in the dimensions of 2.5×2.5×0.3 mm transmits the digital packets containing an ID number and sensing data only when sensing data matche a particular condition. The low-noise force sensing and demonstration of the autonomous transmission from the tactile sensor chip confirm that this sensor is fabricated and designed successfully.

As shown in Table 2, this work has various improvements compared to previously reported device structures. The mechanically formed groove realizes high density through silicon interconnection compared to the traditional TSV or the use of an interposer, which will be important when programming or testing after packaging becomes necessary in the future. More importantly, TSG technology is designed so that backside electrodes are formed after the wafer bonding in the minimum process step. This process enables an ultimately thin package structure that achieves a thinner artificial skin for a robot and a smaller footprint sensor.

The motivation of this research is to solve the realistic and critical problems of an amount of wire and data when the entire robot surface is covered by sensors, which are barely discussed. We believe the device structure and fabrication process presented in this paper can be easily reproduced in most research facilities as it requires only general equipment of the assembly line such as dicing and polishing.

## Figures and Tables

**Table 1:** BCB filling and polishing procedure.

Process	Process Details	
	Spin coating	CYCLOYENE3000-63 3000 rpm × 2 time
BCB Coating	Soft Baking	Hotplate 130 ${}^{\circ }$C for 5 min
	Half-curing	220 ${}^{\circ }$C for 1 h in N${}_{2}$ environment
Polishing	Slurry	Water 800 mL + ${\mathrm{Al}}_{2}O_{3}$ 4g + Triton X-100 2 mL
	Pressure	25 kPa
	Rotation speed	20 rpm
	Polishing pad	UltraPol (Buehler, Lake Bluff, IL, USA)
	Conditioning of polishing pad	6 h with dummy sample with BCB
	Polishing rate	0.75 μm/min
Cleaning	Water polishing	10 min
	Wax removal	Ethanol soaking up to 5 min

**Table 2:** Comparison with the previous report for the MEMS–CMOS surface mountable tactile sensor (${}^{*}$ LTCC: Low Temperature Co-fired Ceramic).

	TSV with Deep RIE [4,5]	LTCC${}^{*}$ Interposer [3]	This Work
Demonstrated TSV	4/5	10	20
Device thickness	650 μm	660 μm	300 μm
Bonding method	Au-Au bonding	Au-Au bonding	BCB bonding
Parasitic capacitance	not reported	3.2 pF	1.33 pF
